# Histopathological changes in septic acute kidney injury in critically ill children: an observational analytical study of postmortem renal biopsies

**DOI:** 10.1186/cc14051

**Published:** 2014-12-03

**Authors:** R Rameshkumar, S Mahadevan, RN Ganesh, P Narayanan, V Bhat

**Affiliations:** 1 Department of Pediatrics, Jawaharlal Institute of Postgraduate Medical Education & Research, Puducherry, India; 2 Department of Pathology, Jawaharlal Institute of Postgraduate Medical Education & Research, Puducherry, India

## Introduction

Critically ill children often manifest acute kidney injury (AKI) as part of their disease process with incidence up to 82% and it is independently associated with poor outcome [1]. Sepsis is the most common contributing factor and little is known about the pathogenesis of septic AKI. There are no consistent histopathological changes in human or experimental septic AKI [2]. Hence, understanding of the structural changes associated with its occurrence is therefore important in the management of AKI in critically ill children.

## Methods

Children aged less than 12 years who died with septic AKI were screened for percutaneous USG-guided kidney biopsy after obtaining written informed consent from July 2012 to June 2014. Three cores of kidney tissue were taken for histopathological evaluation by light, immunofluorescence and electron microscopic examination. Sepsis and AKI was defined using international pediatric sepsis consensus conference and pRIFLE criteria respectively. Events related to death, laboratory parameters and microbiological details 24 hours preceding death were recorded.

## Results

A total of 40 kidney biopsies were done during the study period. Median (IQR) age was 12 (2 to 36) months and PRISM-III was 14 (12 to 18). The most common change was normal histology in 37.5% (*n *= 15) followed by tubular change alone in 22% (*n *= 10), glomerular change alone in 5% (*n *= 2) and blood vessel change in 2.5% (*n *= 1) of specimens. Twelve specimens were showing a combination of changes (tubular + glomerular + interstitium = 5; tubular + glomerular + blood vessels = 3; tubular + glomerular = 2; tubular + interstitium = 1; tubular + glomerular + interstitium + blood vessels = 1). All tubular changes were consistent with acute tubular necrosis (ATN) and changes involved from 5 to 15% of cortex. Thrombotic microangiopathy was diagnosed in 12.5% (*n *= 5) of specimens. There were no significant difference between ATN versus non-ATN groups with respect to baseline characteristics and events related to mortality.

## Conclusion

The most common change in septic AKI in critically ill children is normal histology followed by ATN. This is consistent with published literature [2].
